# Risk of Premenopausal and Postmenopausal Breast Cancer among Multiple Sclerosis Patients

**DOI:** 10.1371/journal.pone.0165027

**Published:** 2016-10-24

**Authors:** Mohammadhossein Hajiebrahimi, Scott Montgomery, Sarah Burkill, Shahram Bahmanyar

**Affiliations:** 1 Clinical Epidemiology Unit, Department of Medicine, Solna, Karolinska Institutet, Stockholm, Sweden; 2 Clinical Epidemiology and Biostatistics, School of Medical Sciences, Örebro University, Örebro, Sweden; 3 Department of Epidemiology and Public Health, University College London, London, United Kingdom; 4 Center for Pharmacoepidemiology & Clinical Epidemiology Unit, Department of Medicine, Karolinska Institutet, Stockholm, Sweden; 5 Department of Public Health, Health Faculty, Golestan University of Medical Sciences, Gorgan, Iran; National Cancer Center, JAPAN

## Abstract

**Objective:**

To investigate risk of premenopausal and postmenopausal breast cancer among Multiple Sclerosis (MS) patients, considering tumor stage.

**Methods:**

The Swedish Patient Register identified 19,330 women with MS between 1968 and 2012, matched individually with a cohort of 193,458 without MS. Matching variables were year of birth, sex, region of residence and vital status at the time of diagnosis. The cancer register identified 471 and 5,753 breast cancer cases among the MS and non-MS cohorts, respectively. Cox proportional hazard models estimated hazard ratios (HR) and 95% confidence intervals (CI) for premenopausal and postmenopausal breast cancer.

**Results:**

Overall risk of postmenopausal breast cancer was 13% higher among MS patients compared with women without MS (HR = 1.13, 95% CI 1.02–1.26). Stratified analyses showed that the risk was statistically significantly increased in women diagnosed between 1968 and 1980 and those who were diagnosed at age 65 or older age. We observed a non-statistically significant risk only for stage 0–1 postmenopausal breast cancer (HR = 1.17, 95% CI 0.93–1.48). MS was not associated with premenopausal breast cancer.

**Conclusion:**

The modest increased risk of postmenopausal breast cancer in women with MS may be due to surveillance bias, where contact with health services for one disease increases the risk of a second diagnosis being recorded.

## Introduction

Multiple Sclerosis is a chronic inflammatory disease of the central nervous system which is more common among the women[1, 2]. Although the overall risk for cancer seems to be lower among MS patients compared with those without MS[3], the risk of some site-specific cancers, such as bladder cancer, is increased among these patients[3, 4]. Findings from previous studies on the association between MS and breast cancer have yielded inconsistent results: the risk of breast cancer was increased [4–7] or there was no association [3, 8, 9]. Moreover, previous studies that looked at tumor size [4] did not take other tumor characteristics into account, such as tumor stage or menopausal status. Severity and prognosis tend to be worse among premenopausal women than postmenopausal women and these subtypes of breast cancer have different risk profiles [10–12].

Using Swedish population registers, we conducted a general population-based cohort study covering the period 1968 to 2012. Our aim was to assess the risk of breast cancer in MS patients, including categorization of breast cancer by menopausal status and tumor stage.

## Materials and Methods

All women who received a diagnosis of MS (primary or underlying diagnosis) in Sweden between 1968 and 2012 were identified, using data from the National Patient Register (N = 19,658). The National Patient Register (NPR) has recorded hospital discharge diagnoses since 1964 with national coverage since 1987 and the current completeness of the register is more than 99%[13]. Since 2001, information is also collected on outpatient visits to hospital (specialist care). The Swedish Cancer Register (SCR) provided data on breast cancer including tumor stage. The register was established in 1958 and collects data on all newly diagnosed cancer in Sweden. The diagnoses are morphologically verified for approximately 98%. Overall underreporting of cancers is estimated as less than 4%[14]. Although the completeness of SCR for some site-specific tumors such as lymphoma is low, the completeness of the register is high for breast cancer [14]. Different editions of International Classification of Diseases (ICD) have been used in the register but all codes are converted to ICD-7 to facilitate assessment of temporal variation. Reporting the data on all newly diagnosed cancer is mandatory for all health providers in Sweden[15]. Highest educational level based on number of years in full-time education (≤9, 10–12 and >12 years) was obtained from the Swedish Register of Education, which has collected data since 1985. The data on emigration and mortality were retrieved from Swedish Total Population Register, which has collected data since 1967 and the Cause of Death Register, which has collected data since 1961 with 100% coverage, respectively. All data were linked using the unique personal identity number assigned to all Swedish residents since 1947[16].

Subjects with MS were individually matched with ten persons without the disease among the general Swedish population by Statistics Sweden (four cases had only 9 controls). The matching criteria were year of birth, sex, region of residence: residential province at the MS diagnosis and vital status at the time of the MS diagnosis (non-MS cohort members were alive when the MS diagnosis was made). Subjects were excluded from the study if breast cancer was diagnosed before MS (n = 275) or the equivalent time in the comparison cohort (n = 2514). As there may be etiological differences for benign and malignant tumors, we investigated only malignant tumors: all benign tumors (50 MS patients and 601 non-MS comparators) and breast cancers that were diagnosed by autopsy (3 MS patients and 3 non-MS comparators) were excluded. The final number of study participants were 19,330 MS patients and 193,458 non-MS women.

Age at cancer diagnosis, using 50 years as the cutoff [17, 18]}, was used to define pre- and postmenopausal breast cancer. We categorized tumor stage in three groups (stage 0–1, 2 and 3–4) instead of the standard classification [19] to have a sufficient number of events for analysis. Tumor stage is defined using three items; T (Tumor size), N (Nodule involvement) and M (Metastasis) and is usually categorized into five subgroups: 0 (carcinoma in situ), 1 (tumors localized to the organ of origin), 2 (tumors localized to the organ of origin with local involvement), 3 (tumor locally extensive spread, particularly to regional lymph nodes), 4 (tumors with distant metastasis).

The study was approved by the research ethics committee of Karolinska Institutet.

### Statistical analysis

We reported event count, person-years, and incidence rates with 95% confidence intervals for the occurrence of all breast cancer diagnoses, as well as divided into premenopausal and postmenopausal breast cancer. For premenopausal women follow up time started from the date of first MS diagnosis registered in the Swedish Patient Register to the death, first migration, premenopausal breast cancer, age 50 or end of study at 31^st^ Dec, 2012 whichever occurred first. We used time from the first diagnosis of MS registered in the Swedish Patient Register to the death, first migration, premenopausal breast cancer, postmenopausal breast cancer or end of study in Dec 31^st^, 2012, whichever occurred first, as the follow up time for postmenopausal breast cancer. Follow up time for the controls started from index date (the date of MS diagnosis in matched cases). Although persons are in fact not at risk for postmenopausal breast cancer before they are 50 years of age, the follow-up was started at the date of MS diagnosis to consider censoring due to other factors e.g. premenopausal breast cancer, emigration or death before age 50 years. We also performed a sensitivity analysis starting the follow-up at age 50 years and observed similar results. Person-years was calculated as the sum of the time difference between the entry dates and censoring events. We also reported incidence rates for categories of age at MS diagnosis and year of MS diagnosis. The 95% CIs for the IRs were calculated assuming that the observed events followed a Poisson distribution [20]. A homogeneity test was performed for age at diagnosis and year of diagnosis.

Cox regression using follow-up years as the underlying time scale was used to estimate HR with 95% CI for premenopausal and postmenopausal breast cancer in women who were diagnosed with MS compared with the non-MS cohort. We adjusted the models for age, sex and region to take into account the matching criteria. The adjusted model provides greater statistical power and similar results to the stratified analysis. We used Cox proportional hazard models, which is a type of survival analysis that takes differences in follow-up time into account. Use of Cox regression gave us the opportunity to model several variables simultaneously and assess the risk through the study period, effectively taking age and other temporal effects into account. The multivariate models included educational level in addition to the matching factors. We categorized the age at MS diagnosis (<18, 18–40, 41–54, 55–64 and ≥65 years) and year of MS diagnosis (1968–1980, 1981–2000 and 2001–2012) and estimated the risk for each subcategory separately. In analyses, stratified by tumor stage (stage 0–1, stage 2, and stage 3–4) the risk of premenopausal and postmenopausal breast cancer for MS patients was estimated. Since information on tumor stage was available from 2000, the data were restricted to events after this time for this sub-analysis using the date of MS diagnosis/index date as the start of follow up time. We investigated possible multiplicative interactions between MS and tumor stage separately in premenopausal- and postmenopausal diagnoses.

We performed additional analyses restricting data to the periods before and after national coverage (from 1987), and the periods of national coverage for hospital discharge data; and with or without outpatient data (from 2001). We have built four different cohorts: MS patients who were diagnosed between 1968 and 1986; MS patients who were diagnosed between 1987 and 2000; the patients who were diagnosed after 2000; and MS patients who were diagnosed between 1987 and 2012. We reanalyzed the data based on these four cohorts. We also performed a sensitivity analysis starting the follow-up time five years before the date of MS diagnosis to account for the potential lag time between age of MS onset and diagnosis [21, 22]. We, moreover, conducted an analysis restricting the data to MS patients who had at least two diagnosis code of MS.

The proportionality assumption was verified for all models, by including a time-by covariate interaction in the model and testing the statistical significance.

All analyses were preformed using SAS software version 9.4 (SAS Institute, NC, and USA)

## Results

Characteristics of the study population are described in Table 1. There were 19,330 patients with MS, and of these 87 were subsequently diagnosed with premenopausal, and 384 with postmenopausal breast cancer. There were 193,458 women without MS in the comparison cohort and of these 942 were diagnosed with premenopausal and 4811 with postmenopausal breast cancer. As MS patients and non-MS comparators were matched on sex, year of birth and region of residence, there were no substantial differences between the cohorts for these variables. Although there is a statistically significant difference (p<0.0001), this may be due to the large sample size, as there were no substantial differences for number of years in full-time education between the two cohorts (Table 1). The homogeneity test showed that there is statistically significant difference in mean of age at diagnosis during the study period (ranged from 41.8 to 49.2 years, p value<0.0001). P-value for age at diagnosis/entry in both premenopausal- and postmenopausal breast cancer were <0.0001.

**Table 1:** Characteristics of MS patients and non-MS matched comparators.

	MS			Non-MS		
	All	Premenopausal breast cancer	Postmenopausal breast cancer	All	Premenopausal breast cancer	Postmenopausal breast cancer
	No (%)^a^	No	No	No (%)^a^	No	No
**Total**^b^	19330 (100)	87	384	193458 (100)	942	4811
**Age at MS diagnosis/entry**						
<18	217 (1.7)	1	0	2167 (1.1)	11	0
18–40	7323 (37.9)	56	65	73143 (37.8)	619	807
41–54	6791 (35.1)	30	175	67898 (35.1)	312	2198
55–64	2830 (14.6)	—	87	28208 (14.6)	—	1148
≥65	2169 (11.2)	—	57	22042 (11.4)	—	658
**Year of MS diagnosis/entry**						
1968–1980	3118 (16.1)	20	123	31177 (16.1)	201	1776
1981–2000	7128 (36.9)	44	183	71427 (36.9)	500	2309
2001–2012	9084 (47.0)	23	78	90854 (47.0)	241	726
**Education, years**						
≤9	5018 (26.0)	19	142	48109 (24.9)	160	1827
10–12	7638 (39.5)	36	116	76721 (39.7)	417	1732
>12	5752 (29.8)	32	101	60021 (31.0)	364	1005
Missing	922 (4.8)	0	25	8607 (4.5)	1	247
P-Value ^c^	<0.0001					

^a^ Column Percent

^b^ P value for homogeneity test for age at diagnosis and year at diagnosis was <0.0001

^c^ p-value for difference between educational levels of MS patients and controls

Table 2 shows the risk associated with having MS for overall-, premenopausal- and postmenopausal breast cancer. The overall incidence rate of breast cancer is 201 and 206 per 100,000 person years for patients with MS and those without MS, respectively. There is no association between MS and overall risk of breast cancer. The incidence rate of premenopausal breast cancer is 81 and 86 per 100,000 person years for patients with MS and those without MS, respectively. There is no association between MS and premenopausal breast cancer. The incidence rate of postmenopausal breast cancer is 163 and 173 per 100,000 person years for patients with MS and those without MS, respectively. Overall, the risk of postmenopausal breast cancer is 13% higher among MS patients, which is statistically significant. Analysis stratified by age at diagnosis/entry shows that the risk of breast cancer is 35% higher and statistically significant for women with a diagnosis of MS recorded at age 65 years or older and that other age groups do not have any increased risk. P-value for year at diagnosis/entry in premenopausal- and postmenopausal breast cancer were 0.02 and 0.16, respectively. Analysis stratified by year of MS diagnosis/entry shows that the increased risk is restricted to women whose MS diagnosis was recorded between 1968 and 1980; with a statistically significant 21%, higher risk of postmenopausal breast cancer. Sensitivity analysis in which the data were restricted to cohort of MS patients who were diagnosed between 1968 and 1986; MS patients who were diagnosed between 1987 and 2000; the patients who were diagnosed after 2000; and MS patients who were diagnosed between 1987 and 2012 showed that the results are robust and in particular, the risk of postmenopausal breast cancer among MS patients diagnosed during different periods is not substantially altered (S1–S8 Tables). The results for women diagnosed with MS at age 65 years or older showed a 40%, 40% and 21% percent increased risk of postmenopausal breast cancer in the periods of MS diagnosis (1968–1986, 1987–2000, 2001–2012), respectively, although the difference is not statistically significant (S1, S3 and S5 Tables). The results of sensitivity analysis starting follow-time five years before the date of first recorded MS diagnosis were shown in S9 and S10 Tables. It has been shown that the risk of premenopausal breast cancer is 40% higher when their MS were diagnosed between 1968 and 1980, statistically insignificant. Analysis restricted to MS patients who had at least two diagnoses of MS (S11 Table) did not change notably compared with the main analysis

**Table 2:** Incidence rate, Hazard ratios (HR) and 95% confidence intervals (CI) for association between MS and breast cancer, stratified by menopausal status.

	MS				Non-MS				Unadjusted	Adjusted^a^
	Number	Person years (PY)	Event (%)^b^	Incidence rate per 100,000 PY(95% CI)	Number	Person Years (PY)	Event (%)^b^	Incidence rate per 100,000 PY(95% CI)	HR (95% CI)	HR (95% CI)
**Total** ^c^	19330	236114	474 (2.5)	201 (183–219)	193461	2788816	5756 (3.0)	206 (201–212)	1.02 (0.93–1.12)	1.08 (0.98–1.19)
**Premenopausal women**										
**Total**	12484	107778	87 (0.7)	81 (65–99)	124833	1101490	942 (0.8)	86 (80–91)	0.95 (0.76–1.18)	0.96 (0.77–1.19)
**Age at MS diagnosis/entry** ^d^										
<18	217	3577	1 (0.5)	28 (3–130)	2167	37428	11 (0.5)	29 (16–51)	1.01 (0.13–7.81)	0.90 (0.11–7.00)
18–40	7323	81579	56 (0.8)	69 (52–88)	73143	833896	619 (0.8)	74 (69–80)	0.94 (0.71–1.23)	0.94 (0.72–1.24)
41–50	4944	22621	30 (0.6)	133 (91–187)	49523	230166	312 (0.6)	136 (121–151)	0.98 (0.67–1.43)	0.99 (0.68–1.43)
**Year of MS diagnosis/entry**^e^										
1968–1980	1847	23694	20 (1.1)	84 (69–91)	18486	252330	201 (1.1)	80 (69–91)	1.07 (0.68–1.70)	1.10 (0.69–1.74)
1981–2000	4566	52307	44 (1.0)	84 (86–102)	45707	532704	500 (1.1)	94 (86–102)	0.90 (0.66–1.23)	0.91 (0.67–1.24)
2001–2012	6071	31778	23 (0.4)	72 (67–86)	60640	316456	241 (0.4)	76 (67–86)	0.95 (0.62–1.46)	0.96 (0.62–1.47)
**Postmenopausal women**										
**Total**	19330	236091	384 (2.0)	163 (147–180)	193461	2788779	4811 (2.5)	173 (168–177)	1.01 (0.91–1.12)	1.13 (1.02–1.26)
**Age at MS diagnosis/entry**^f^										
<18	217	3643	0 (0.0)	0 (0.0)	2167	38459	0 (0.0)	0 (0.0)	---	---
18–40	7323	102454	65 (0.9)	63(49–80)	73143	1104959	807 (1.1)	73 (68–78)	1.03 (0.80–1.33)	1.03 (0.80–1.32)
41–54	6791	86013	175 (2.6)	203(175–235)	67898	1033889	2198 (3.2)	213 (204–222)	1.05 (0.90–1.22)	1.05 (0.90–1.22)
55–64	2830	29552	87 (3.1)	294(237–361)	28208	389210	1148 (4.1)	295 (278–312)	1.02 (0.82–1.27)	1.02 (0.82–1.26)
≥65	2169	14428	57 (2.6)	395(302–508)	22042	222264	658 (3.0)	296 (274–319)	1.36 (1.04–1.78)	1.35 (1.03–1.77)
**Year of MS diagnosis/entry**^g^										
1968–1980	3118	63948	123 (3.9)	192(161–229)	31177	891853	1776 (5.7)	199 (190–209)	1.07 (0.89–1.28)	1.21 (1.01–1.45)
1981–2000	7128	116165	183 (2.6)	158(136–182)	71427	1328218	2309 (3.2)	174 (167–181)	0.94 (0.81–1.89)	1.06 (0.91–1.23)
2001–2012	9084	55979	78 (0.9)	139(111–173)	90854	568708	726 (0.8)	128 (119–137)	1.09 (0.87–1.38)	1.15 (0.91–1.45)

^a^ Adjusted for age at MS diagnosis, residential location and educational level

^b^ Percent of women who were diagnosed with breast cancer among MS and non-MS women

^c^ Breast cancer tumors diagnosed through an autopsy are included

^d^ P value for difference between the categories in the adjusted model: <0.0001

^e^ P value for difference between the categories in the adjusted model: <0.02

^f^ P value difference between the categories in the adjusted model: <0.0001

^g^ P value for difference between the categories in the adjusted model: 0.16

Table 3 displays the risk of premenopausal- and postmenopausal breast cancer among women with and without MS, stratified by tumor stage. There is no association between MS and premenopausal breast cancer. There is no statistically significant interaction between MS and premenopausal breast cancer tumor stage (P = 0.38). MS patients have a 17% higher risk of stage 0–1 postmenopausal breast cancer, which does not achieve statistical significance (HR: 1.17, 95% CI 0.93–1.48). The interaction between MS and postmenopausal breast cancer tumor stage is not statistically significant, as well (P = 0.57). Analysis stratified by year of MS diagnosis showed that risk of stage 0–1 postmenopausal breast cancer was statistically significant 50% higher among women with MS diagnosed during 2001–2012 (S6 Table).

**Table 3:** Incidence Rate, Hazard ratios (HR) and 95% confidence intervals (CI) for association between MS and breast cancer, stratified by stage of cancer and menopausal status.

	MS				Non-MS				Unadjusted	Adjusted ^a^
	Number	Person years (PY)	Event (%)	Incidence Rate per 100,000 PY (95% CI)	Number	Person years (PY)	Event (%)	Incidence Rate per 100,000 PY (95% CI)	HR (95% CI)	HR (95% CI)
**Premenopausal women**										
**Total**	12484	107969	35 (0.3)	32 (23–45)	124833	1103882	386 (0.3)	35 (32–39)	0.94 (0.66–1.32)	0.94 (0.67–1.33)
**Stage**										
0–1	12484	108037	15 (0.1)	14 (8–22)	124833	1104549	172 (0.1)	16 (13–18)	0.90 (0.53–1.52)	0.91 (0.54–1.54)
2	12484	108026	17 (0.1)	16 (10–25)	124833	1104531	186 (0.2)	17 (15–19)	0.94 (0.57–1.55)	0.95 (0.58–1.55)
3–4	12484	108066	3 (0.0)	3 (1–7)	124833	1105013	28 (0.0)	3 (2–4)	1.11 (0.34–3.67)	1.10 (0.34–3.62)
P for Interaction										0.38
**Postmenopausal women**										
**Total**	19330	238306	143 (0.7)	60 (51–71)	193458	2824287	1882(1.0)	67 (64–70)	1.00 (0.85–1.19)	1.07 (0.90–1.27)
**Stage**										
0–1	19330	238510	80 (0.4)	34 (27–42)	193458	2827825	929 (0.5)	33 (31–35)	1.13 (0.90–1.42)	1.17 (0.93–1.48)
2	19330	238631	57 (0.3)	24 (18–31)	193458	2828777	798 (0.4)	28 (26–30)	0.94 (0.72–1.24)	1.03 (0.78–1.34)
3–4	19330	238828	6 (0.0)	3 (1–5)	193458	2831922	155 (0.1)	5 (5–6)	0.56 (0.25–1.26)	0.64 (0.28–1.45)
P for Interaction										0.57

^a^ Adjusted for age at MS diagnosis, residential location, duration of the MS and educational level

## Discussion

This general population-based cohort study found an apparent risk increase for postmenopausal breast cancer, mainly stage 0–1, associated with MS but only among women with an MS diagnosis recorded at an older age. There was no association between MS and premenopausal breast cancers. We observed that the incidence rate of breast cancer in women without MS is 206 per 100,000 person-years which is consistent results from previous reports[23].

Increased risk of breast cancer among MS patients was shown by some studies [4–7] but not all [3, 8, 9]. However, none of previous studies investigated risk of premenopausal- and postmenopausal breast cancer separately. Estimating breast cancer risk based on menopausal status is important because these are considered as two distinct diseases[24]. It has been shown that premenopausal breast cancers are more severe, more receptor negative and with worse prognosis, while postmenopausal breast cancers have higher receptor positive tumor rates and a better prognosis [10, 25–28]. Also, no previous study has investigated tumor stage when evaluating the association between MS and breast cancer risk. If symptoms of cancer are misinterpreted as those of multiple sclerosis, this would lead to a delay in its diagnosis which could result in a higher tumor stage at diagnosis. On the other hand, if there is surveillance bias, the tumors may be diagnosed earlier with a lower stage. Only two studies that we are aware of [4, 29] investigated tumor size and found that breast cancer tumors were larger in MS patients than in women without MS. Adjusting for parity and age at first pregnancy did not change their results notably. In our study, the observed increased risk of postmenopausal breast cancer with low tumor stage in patients with MS, is restricted to women who were diagnosed with MS at an older age. The results showed the higher risk even after stratifying the data on the age at MS diagnosis (results did not show). Increased risk of postmenopausal breast cancer with low tumor stage might be attributed to surveillance bias as MS patients are followed and monitored by medical clinics more frequent and tumors may be detected at an earlier stage. Further support for this speculation is that the increased risk of postmenopausal breast cancer is observed particularly during the earliest time period of the study: the diagnosis of MS recorded in the Patient Register may not be the first diagnosis the patient received, but we cannot identify earlier diagnoses as the register did not cover this period. The increased risk of postmenopausal breast cancer can also be due to lower parity in women with MS[5]. However, we cannot rule out the possibility of chance finding.

The results from our study show that the risk of breast cancer among MS patients is higher among patients who were diagnosed with MS at age 65 or older. Some previous studies [30, 31] have reported that the risk of cancer is increased among MS patients who have been treated for their disease. Our results argue against a major influence of MS therapies on risk of breast cancer. Our findings, moreover, show that the risk of postmenopausal breast cancer is 21% higher among MS patients who were diagnosed in years between 1968 and 1980 but not in other subgroups. The increased risk of postmenopausal breast cancer during this period of time might be due to influence of prevalence cases.

Strengths of this study are the large sample size and the general population-based cohort design. Using Swedish national registers gave us an opportunity to have adequate information and enough statistical power to investigate the risk of premenopausal—and postmenopausal breast cancer among MS patients, taking stage of tumor into account. This study has also some potential limitations. Restricting the data to the date between 2000 and 2012 limited our statistical power. For this reason, we categorized tumor stage in three groups instead of using the standard classification[19]. Another caveat is a lack of information on age of onset. However, we performed a sensitivity analysis starting the follow-up five years before the date of MS diagnosis, which showed that our results are robust. As the Swedish National Patient Register collected data only on inpatient diagnoses until 2001 and outpatients subsequently, we only have information on the first recorded diagnosis, rather than the first diagnosis received by the patient. Therefore, we conducted a sensitivity analysis including women who were diagnosed after 2001. Like other register-based studies, we did not have information on some potentially important disease specific variables such as MS disease course and severity, MS treatment and information about number of familial cancers. In addition, since the education register started since 1985, those who were censored before 1985 had missing information for education. Finally, we did not have information on some potential confounding factors such as parity, or hormone replacement therapy.

In conclusion, patients with MS may have a moderately increased risk of postmenopausal breast cancer, but this might be due to surveillance bias rather than reflecting a true risk. MS does not appear to be associated with premenopausal breast cancer.

## Supporting Information

S1 Table Incidence rate, Hazard ratios (HR) and 95% confidence intervals (CI) for association between MS, diagnosed between 1968 and 1986, and breast cancer, stratified by menopausal status. (DOCX)

S2 Table Incidence Rate, Hazard ratios (HR) and 95% confidence intervals (CI) for association between MS, diagnosed between 1968 and 1986, and breast cancer, stratified by stage of cancer and menopausal status. (DOCX)

S3 Table Incidence rate, Hazard ratios (HR) and 95% confidence intervals (CI) for association between MS, diagnosed between 1987 and 2000, and breast cancer, stratified by menopausal status. (DOCX)

S4 Table Incidence Rate, Hazard ratios (HR) and 95% confidence intervals (CI) for association between MS, diagnosed between 1987 and 2000, and breast cancer, stratified by stage of cancer and menopausal status. (DOCX)

S5 Table Incidence rate, Hazard ratios (HR) and 95% confidence intervals (CI) for association between MS, diagnosed between 2001 and 2012, and breast cancer, stratified by menopausal status. (DOCX)

S6 Table Incidence Rate, Hazard ratios (HR) and 95% confidence intervals (CI) for association between MS, diagnosed between 2001 and 2012, and breast cancer, stratified by stage of cancer and menopausal status. (DOCX)

S7 Table Incidence rate, Hazard ratios (HR) and 95% confidence intervals (CI) for association between MS, diagnosed between 1987 and 2012, and breast cancer, stratified by menopausal status. (DOCX)

S8 Table Incidence Rate, Hazard ratios (HR) and 95% confidence intervals (CI) for association between MS, diagnosed between 1987 and 2012, and breast cancer, stratified by stage of cancer and menopausal status. (DOCX)

S9 Table Incidence rate, Hazard ratios (HR) and 95% confidence intervals (CI) for association between MS and breast cancer, stratified by menopausal status (follow up time since five years before the date of first registration). (DOCX)

S10 Table Incidence Rate, Hazard ratios (HR) and 95% confidence intervals (CI) for association between MS and breast cancer, stratified by stage of cancer and menopausal status, (follow up time since five years before the date of first registration). (DOCX)

S11 Table Incidence rate, Hazard ratios (HR) and 95% confidence intervals (CI) for association between MS (Those patients who had two codes of MS) and breast cancer, stratified by menopausal status. (DOCX)
