# Supplementary material for: Risk of Premenopausal and Postmenopausal Breast Cancer among Multiple Sclerosis Patients
Source: PLoS One. 2016 Oct 24;11(10):e0165027. doi: 10.1371/journal.pone.0165027 (PMC5077134; doi:10.1371/journal.pone.0165027)
Supplement: S1 Table — (DOCX) [file pone.0165027.s001.docx]

|  | **MS** | | | | | **Non-MS** | | | | **Unadjusted** | **Adjusted ^a^** |
| --- | --- | --- | --- | --- | --- | --- | --- | --- | --- | --- | --- |
|  | **Number** | **Person years (PY)** | **Event (%)** | **Incidence rate per 100,000 PY**  **(95% CI)** | **Number** | | **Person**  **Years (PY)** | **Event (%)** | **Incidence rate per 100,000 PY**  **(95% CI)** | **HR (95% CI)** | **HR (95% CI)** |
| **Total** | 5099 | 101713 | 214 | 210 (184-240) | 50973 | | 1358814 | 3019 | 222 (214-230) | 1.01 (0.88-1.16) | 1.08 (0.94-1.24) |
| **Premenopausal women** | |  |  |  |  | |  |  |  |  |  |
| **Total** | 2970 | 38097 | 29 (1.0) | 76 (52-108) | 29762 | | 402914 | 334 (1.1) | 83 (74-92) | 0.93 (0.64-1.36) | 0.94 (0.64-1.36) |
| **Age at MS diagnosis/entry** | |  |  |  |  | |  |  |  |  |  |
| <18 | 71 | 2073 | 1 (1.4) | 48 (4-225) | 707 | | 21972 | 11 (1.6) | 50 (27-87) | 1.01 (0.13-7.79) | 0.90 (0.12-7.03) |
| 18-40 | 1726 | 29957 | 19 (1.1) | 63 (39-97) | 17229 | | 317392 | 247 (1.4) | 78 (69-88) | 0.83 (0.52-1.33) | 0.83 (0.52-1.32) |
| 41-50 | 1173 | 6067 | 9 (0.8) | 148 (73-271) | 11826 | | 63550 | 76 (0.7) | 120 (95-149) | 1.25 (0.63-2.50) | 1.28 (0.64-2.55) |
| **Postmenopausal women** | |  |  |  |  | |  |  |  |  |  |
| **Total** | 5099 | 101713 | 185 (3.6) | 182 (157-210) | 50973 | | 1358814 | 2685 (5.2) | 198 (190-205) | 1.00 (0.86-1.16) | 1.13 (0.98-1.32) |
| **Age at MS diagnosis/entry** | |  |  |  |  | |  |  |  |  |  |
| <18 | 71 | 2139 | 0 (0.0) | 0 (0.0) | 707 | | 23003 | 0 (0.0) | 0 (0.0) | ---- | ---- |
| 18-40 | 1726 | 46826 | 53 (3.1) | 113(86-147) | 17229 | | 544281 | 706 (4.1) | 130(120-140) | 0.99 (0.75-1.31) | 0.99 (0.75-1.31) |
| 41-54 | 1673 | 34311 | 76 (4.5) | 222(176-276) | 16745 | | 490221 | 1126 (6.7) | 230(217-243) | 1.09 (0.86-1.38) | 1.09 (0.90-1.38) |
| 55-64 | 876 | 12817 | 34 (3.9) | 265(187-366) | 8769 | | 202484 | 8769 (6.4) | 279(257-303) | 1.01 (0.71-1.43) | 1.01 (0.71-1.43) |
| ≥65 | 753 | 5621 | 22 (2.9) | 391(252-582) | 7523 | | 98826 | 288 (3.8) | 291(259-327) | 1.41 (0.91-2.17) | 1.40 (0.91-2.17) |

S1: Incidence rate, Hazard ratios (HR) and 95% confidence intervals (CI) for association between MS, diagnosed between 1968 and 1986, and breast cancer, stratified by menopausal status

^a^ Adjusted for age at MS diagnosis, residential location
