# Supplementary material for: Risk of Premenopausal and Postmenopausal Breast Cancer among Multiple Sclerosis Patients
Source: PLoS One. 2016 Oct 24;11(10):e0165027. doi: 10.1371/journal.pone.0165027 (PMC5077134; doi:10.1371/journal.pone.0165027)
Supplement: S2 Table — (DOCX) [file pone.0165027.s002.docx]

S2: Incidence Rate, Hazard ratios (HR) and 95% confidence intervals (CI) for association between MS, diagnosed between 1968 and 1986, and breast cancer, stratified by stage of cancer and menopausal status.

^a^ Adjusted for age at MS diagnosis, residential location, duration of the MS

|  | **MS** | | | | | **Non-MS** | | | | | | | **Unadjusted** | | **Adjusted ^a^** | |
| --- | --- | --- | --- | --- | --- | --- | --- | --- | --- | --- | --- | --- | --- | --- | --- | --- |
|  | **Number** | **Person years (PY)** | **Event (%)** | **Incidence Rate per 100,000 PY**  **(95% CI)** | | **Number** | | **Person years (PY)** | **Event (%)** | **Incidence Rate per 100,000 PY**  **(95% CI)** | | | **HR (95% CI)** | | **HR (95% CI)** | |
| **Premenopausal women** | |  | |  | |  | |  |  |  | | |  | |  | |
| **Total** | 2970 | 38185 | 2 (0.1) | 5 (1-17) | | 29762 | | 404155 | 38 (0.1) | 9 (7-13) | | | 0.59 (0.14-2.46) | | 0.58 (0.14-2.42) | |
| Stage |  |  |  |  | |  | |  |  |  | | |  | |  | |
| 0-1 | 2970 | 38187 | 1 (0.0) | 3 (0-12) | | 29762 | | 404225 | 16 (0.1) | 4 (2-6) | | | 0.71 (0.09-5.32) | | 0.69 (0.09-5.24) | |
| 2 | 2970 | 38189 | 1 (0.0) | 3 (0-12) | | 29762 | | 404197 | 18 (0.1) | 4 (3-7) | | | 0.62 (0.08-4.64) | | 0.63 (0.08-4.72) | |
| 3-4 | 2970 | 38190 | 0 (0.0) | 0 | | 29762 | | 404250 | 4 (0.0) | 1 (0-2) | | | 0.00 | | 0.00 | |
| P for Interaction |  |  |  |  | |  | |  |  |  | | |  | | 0.50 | |
| **Postmenopausal women** | |  | |  | |  | |  |  |  | | |  | |  | |
| **Total** | 5099 | 103129 | 35 (0.7) | | 34 (24-47) | | 50973 | 1384529 | 702 (1.4) | | 51 (47-55) | | | 0.88 (0.63-1.24) | | 0.92 (0.65-1.29) |
| Stage |  |  |  | |  | |  |  |  | | |  | |  | |  |
| 0-1 | 5099 | 103191 | 15 (0.3) | | 15 (8-23) | | 50973 | 1385924 | 339 (0.7) | 24 (22-27) | | | | 0.77 (0.46-1.29) | | 0.76 (0.45-1.27) |
| 2 | 5099 | 103190 | 18 (0.4) | | 17 (11-27) | | 50973 | 1386454 | 295 (0.6) | 21 (19-24) | | | | 1.10 (0.68-1.77) | | 1.21 (0.75-1.95) |
| 3-4 | 5099 | 103251 | 2 (0.0) | | 2 (0-6) | | 50973 | 1387651 | 68 (0.1) | 5 (4-6) | | | | 0.56 (0.14-2.30) | | 0.71 (0.17-2.89) |
| P for Interaction |  |  |  | |  | |  |  |  |  | | | |  | | 0.92 |
