# Supplementary material for: Risk of Premenopausal and Postmenopausal Breast Cancer among Multiple Sclerosis Patients
Source: PLoS One. 2016 Oct 24;11(10):e0165027. doi: 10.1371/journal.pone.0165027 (PMC5077134; doi:10.1371/journal.pone.0165027)
Supplement: S3 Table — (DOCX) [file pone.0165027.s003.docx]

S3: Incidence rate, Hazard ratios (HR) and 95% confidence intervals (CI) for association between MS, diagnosed between 1987 and 2000, and breast cancer, stratified by menopausal status

^a^ Adjusted for age at MS diagnosis, residential location and educational level.

|  | **MS** | | | | | **Non-MS** | | | | **Unadjusted** | **Adjusted ^a^** |
| --- | --- | --- | --- | --- | --- | --- | --- | --- | --- | --- | --- |
|  | **Number** | **Person years (PY)** | **Event (%)** | **Incidence rate per 100,000 PY**  **(95% CI)** | **Number** | | **Person**  **Years (PY)** | **Event (%)** | **Incidence rate per 100,000 PY**  **(95% CI)** | **HR (95% CI)** | **HR (95% CI)** |
| **Total** | 5147 | 78399 | 156 | 199 (170-232) | 51631 | | 861258 | 1767 | 205 (196-215) | 0,98 (0.84-1.16) | 1.04 (0.88-1.22) |
| **Premenopausal women** | |  |  |  |  | |  |  |  |  |  |
| **Total** | 3443 | 37904 | 35 (1.0) | 92 (65-127) | 34431 | | 382121 | 367 (1.1) | 96 (87-106) | 0.96 (0.68-1.36) | 0.97 (0.68-1.37) |
| **Age at MS diagnosis/entry** | |  |  |  |  | |  |  |  |  |  |
| <18 | 56 | 930 | 0 (0.0) | 0 (0.0) | 560 | | 9702 | 0 (0.0) | 0 (0.0) | -- | -- |
| 18-40 | 1951 | 29617 | 27 (1.3) | 91 (61-131) | 19482 | | 297996 | 259 (1.3) | 87 (77-98) | 1.05 (0.71-1.57) | 1.06 (0.71-1.57) |
| 41-50 | 1436 | 7356 | 8 (0.6) | 108 (51-205) | 14389 | | 74422 | 108 (0.8) | 145 (120-174) | 0.75 (0.37-1.54) | 0.75 (0.37-1.54) |
| **Postmenopausal women** | |  |  |  |  | |  |  |  |  |  |
| **Total** | 5147 | 78399 | 121 (2.4) | 154 (129-184) | 51631 | | 861258 | 1400 (2.7) | 163 (154-171) | 0.97 (0.81-1.17) | 1.09 (0.90-1.31) |
| **Age at MS diagnosis/entry** | |  |  |  |  | |  |  |  |  |  |
| <18 | 56 | 930 | 0 (0.0) | 0 (0.0) | 560 | | 9702 | 0 (0.0) | 0 (0.0) | ---- | ---- |
| 18-40 | 1951 | 33610 | 12 (0.6) | 35 (19-60) | 19482 | | 342045 | 101 (0.5) | 30 (24-36) | 1.26 (0.70-2.30) | 1.26 (0.69-2.29) |
| 41-54 | 1933 | 31040 | 68 (3.5) | 219 (172-276) | 19387 | | 335898 | 786 (4.1) | 234 (218-251) | 0.97 (0.75-1.24) | 0.96 (0.75-1.24) |
| 55-64 | 596 | 8035 | 23 (3.9) | 286 (186-422) | 5926 | | 96724 | 301 (5.6) | 311 (278-348) | 0.92 (0.60-1.41) | 0.92 (0.60-1.40) |
| ≥65 | 611 | 4785 | 18 (3.0) | 376 (231-582) | 6276 | | 76888 | 212 (3.4) | 276 (240-315) | 1.39 (0.86-2.26) | 1.40 (0.86-2.27) |
