# Supplementary material for: Risk of Premenopausal and Postmenopausal Breast Cancer among Multiple Sclerosis Patients
Source: PLoS One. 2016 Oct 24;11(10):e0165027. doi: 10.1371/journal.pone.0165027 (PMC5077134; doi:10.1371/journal.pone.0165027)
Supplement: S4 Table — (DOCX) [file pone.0165027.s004.docx]

S4: Incidence Rate, Hazard ratios (HR) and 95% confidence intervals (CI) for association between MS, diagnosed between 1987 and 2000, and breast cancer, stratified by stage of cancer and menopausal status.

^a^ Adjusted for age at MS diagnosis, residential location, duration of the MS and educational level.

|  | **MS** | | | | | **Non-MS** | | | | | | | **Unadjusted** | | **Adjusted ^a^** | |
| --- | --- | --- | --- | --- | --- | --- | --- | --- | --- | --- | --- | --- | --- | --- | --- | --- |
|  | **Number** | **Person years (PY)** | **Event (%)** | **Incidence Rate per 100,000 PY**  **(95% CI)** | | **Number** | | **Person years (PY)** | **Event (%)** | **Incidence Rate per 100,000 PY**  **(95% CI)** | | | **HR (95% CI)** | | **HR (95% CI)** | |
| **Premenopausal women** | |  | |  | |  | |  |  |  | | |  | |  | |
| **Total** | 3443 | 37979 | 16 (0.5) | 42 (25-67) | | 34431 | | 383087 | 156 (0.5) | 41 (35-47) | | | 1.04 (0.62-1.74) | | 1.06 (0.64-1.78) | |
| Stage |  |  |  |  | |  | |  |  |  | | |  | |  | |
| 0-1 | 3443 | 38020 | 5 (0.2) | 13 (5-29) | | 34431 | | 383367 | 75 (0.2) | 20 (16-24) | | | 0.68 (0.27-1.67) | | 0.69 (0.28-1.72) | |
| 2 | 3443 | 38007 | 8 (0.2) | 21 (10-40) | | 34431 | | 383423 | 69 (0.2) | 18 (14-23) | | | 1.18 (0.57-2.45) | | 1.20 (0.58-2.50) | |
| 3-4 | 3443 | 38021 | 3 (0.1) | 8 (2-21) | | 34431 | | 383607 | 12 (0.0) | 3 (2-5) | | | 2.54 (0.72-8.99) | | 2.54 (0.72-9.01) | |
| P for Interaction |  |  |  |  | |  | |  |  |  | | |  | | 0.93 | |
| **Postmenopausal women** | |  | |  | |  | |  |  |  | | |  | |  | |
| **Total** | 5147 | 79170 | 50 (1.0) | | 63 (47-83) | | 51631 | 870973 | 657 (1.3) | | 75 (70-81) | | | 0.88 (0.66-1.17) | | 0.98 (0.73-1.31) |
| Stage |  |  |  | |  | |  |  |  | | |  | |  | |  |
| 0-1 | 5147 | 79244 | 29 (0.6) | | 37 (25-52) | | 51631 | 872182 | 337 (0.7) | 39 (35-43) | | | | 1.00 (0.68-1.46) | | 1.09 (0.75-1.59) |
| 2 | 5147 | 79313 | 18 (0.4) | | 23 (14-35) | | 51631 | 872554 | 269 (0.5) | 31 (28-35) | | | | 0.77 (0.48-1.25) | | 0.87 (0.54-1.41) |
| 3-4 | 5147 | 79381 | 3 (0.1) | | 4 (1-10) | | 51631 | 873656 | 51 (0.1) | 6 (4-8) | | | | 0.70 (0.22-2.25) | | 0.85 (0.27-2.74) |
| P for Interaction |  |  |  | |  | |  |  |  |  | | | |  | | 0.40 |
