# Supplementary material for: Risk of Premenopausal and Postmenopausal Breast Cancer among Multiple Sclerosis Patients
Source: PLoS One. 2016 Oct 24;11(10):e0165027. doi: 10.1371/journal.pone.0165027 (PMC5077134; doi:10.1371/journal.pone.0165027)
Supplement: S5 Table — (DOCX) [file pone.0165027.s005.docx]

S5: Incidence rate, Hazard ratios (HR) and 95% confidence intervals (CI) for association between MS, diagnosed between 2001 and 2012, and breast cancer, stratified by menopausal status

^a^ Adjusted for age at MS diagnosis, residential location and educational level.

|  | **MS** | | | | | **Non-MS** | | | | **Unadjusted** | **Adjusted ^a^** |
| --- | --- | --- | --- | --- | --- | --- | --- | --- | --- | --- | --- |
|  | **Number** | **Person years (PY)** | **Event (%)** | **Incidence rate per 100,000 PY**  **(95% CI)** | **Number** | | **Person**  **Years (PY)** | **Event (%)** | **Incidence rate per 100,000 PY**  **(95% CI)** | **HR (95% CI)** | **HR (95% CI)** |
| **Total** | 9084 | 55979 | 101 | 180 (148-218) | 90854 | | 568708 | 967 | 170 (160-181) | 1.06 (0.87-1.30) | 1.09 (0.88-1.33) |
| **Premenopausal women** | |  |  |  |  | |  |  |  |  |  |
| **Total** | 6071 | 31778 | 23 (0.4) | 72 (47-107) | 60640 | | 316456 | 241 (0.4) | 76 (67-86) | 0.95 (0.62-1.46) | 0.95 (0.62-1.46) |
| **Age at MS diagnosis/entry** | |  |  |  |  | |  |  |  |  |  |
| <18 | 90 | 575 | 0 (0.0) | 0 (0.0) | 900 | | 5754 | 0 (0.0) | 0 (0.0) | -- | -- |
| 18-40 | 3646 | 22005 | 10 (0.3) | 45 (23-81) | 36432 | | 218508 | 113 (0.3) | 52 (43-62) | 0.88 (0.46-1.68) | 0.88 (0.46-1.68) |
| 41-50 | 2335 | 9198 | 13 (0.6) | 141 (79-235) | 23308 | | 92193 | 128 (0.6) | 139 (116-164) | 1.01 (0.57-1.80) | 1.02 (0.38-1.81) |
| **Postmenopausal women** | |  |  |  |  | |  |  |  |  |  |
| **Total** | 9084 | 55979 | 78 (0.9) | 139 (111-173) | 90854 | | 568709 | 726 (0.9) | 128 (119-137) | 1.09 (0.87-1.38) | 1.15 (0.91-1.45) |
| **Age at MS diagnosis/entry** | |  |  |  |  | |  |  |  |  |  |
| <18 | 90 | 575 | 0 (0.0) | 0 (0.0) | 900 | | 5754 | 0 (0.0) | 0 (0.0) | ---- | ---- |
| 18-40 | 3646 | 22018 | 0 (0.0) | 0 (0.0) | 36432 | | 218633 | 0 (0.0) | 0 (0.0) | ---- | ---- |
| 41-54 | 3185 | 20662 | 31 (1.0) | 150 (104-210) | 31766 | | 207769 | 286 (0.9) | 138 (122-154) | 1.09 (0.76-1.58) | 1.09 (0.75-1.58) |
| 55-64 | 1358 | 8701 | 30 (2.2) | 344 (237-485) | 13513 | | 90001 | 282 (2.1) | 313 (278-352) | 1.10 (0.76-1.61) | 1.10 (0.75-1.60) |
| ≥65 | 805 | 4023 | 17 (2.1) | 423 (256-661) | 8243 | | 46550 | 158 (1.9) | 339 (290-396) | 1.24 (0.75-2.04) | 1.21 (0.74-2.00) |
