# Supplementary material for: Risk of Premenopausal and Postmenopausal Breast Cancer among Multiple Sclerosis Patients
Source: PLoS One. 2016 Oct 24;11(10):e0165027. doi: 10.1371/journal.pone.0165027 (PMC5077134; doi:10.1371/journal.pone.0165027)
Supplement: S6 Table — (DOCX) [file pone.0165027.s006.docx]

S6: Incidence Rate, Hazard ratios (HR) and 95% confidence intervals (CI) for association between MS, diagnosed between 2001 and 2012, and breast cancer, stratified by stage of cancer and menopausal status.

^a^ Adjusted for age at MS diagnosis, residential location, duration of the MS and educational level.

|  | **MS** | | | | | **Non-MS** | | | | | | | **Unadjusted** | | **Adjusted ^a^** | |
| --- | --- | --- | --- | --- | --- | --- | --- | --- | --- | --- | --- | --- | --- | --- | --- | --- |
|  | **Number** | **Person years (PY)** | **Event (%)** | **Incidence Rate per 100,000 PY**  **(95% CI)** | | **Number** | | **Person years (PY)** | **Event (%)** | **Incidence Rate per 100,000 PY**  **(95% CI)** | | | **HR (95% CI)** | | **HR (95% CI)** | |
| **Premenopausal women** | |  | |  | |  | |  |  |  | | |  | |  | |
| **Total** | 6071 | 31804 | 17 (0.3) | 53 (32-84) | | 60640 | | 316640 | 192 (0.3) | 61 (53-70) | | | 0.88 (0.54-1.45) | | 0.89 (0.54-1.45) | |
| Stage |  |  |  |  | |  | |  |  |  | | |  | |  | |
| 0-1 | 6071 | 31829 | 9 (0.2) | 28 (14-52) | | 60640 | | 316958 | 81 (0.1) | 26 (20-32) | | | 1.11 (0.56-2.20) | | 1.11 (0.56-2.22)) | |
| 2 | 6071 | 31830 | 8 (0.1) | 25 (12-47) | | 60640 | | 316912 | 99 (0.2) | 31 (26-38) | | | 0.80 (0.39-1.65) | | 0.81 (0.39-1.66) | |
| 3-4 | 6071 | 31855 | 0 (0.0) | 0 (0.0) | | 60640 | | 317156 | 12 (0.0) | 4 (2-6) | | | --- | | --- | |
| P for Interaction |  |  |  |  | |  | |  |  |  | | |  | | 0.25 | |
| **Postmenopausal women** | |  | |  | |  | |  |  |  | | |  | |  | |
| **Total** | 9084 | 56171 | 58 (0.6) | | 103 (79-132) | | 90854 | 570643 | 523 (0.6) | | 92 (84-100) | | | 1.13 (0.86-1.48) | | 1.18 (0.90-1.55) |
| Stage |  |  |  | |  | |  |  |  | | |  | |  | |  |
| 0-1 | 9084 | 56239 | 36 (0.4) | | 64 (46-88) | | 90854 | 571576 | 253 (0.3) | 44 (39-50) | | | | 1.45 (1.02-2.06) | | 1.50 (1.06-2.14) |
| 2 | 9084 | 56292 | 21 (0.2) | | 37 (24-56) | | 90854 | 571626 | 234 (0.3) | 41 (36-46) | | | | 0.91 (0.58-1.43) | | 0.96 (0.62-1.51) |
| 3-4 | 9084 | 56360 | 1 (0.0) | | 2 (0-8) | | 90854 | 572473 | 36 (0.0) | 6 (4-9) | | | | 0.28 (0.04-2.07) | | 0.31 (0.04-2.26) |
| P for Interaction |  |  |  | |  | |  |  |  |  | | | |  | | 0.82 |
