# Supplementary material for: Risk of Premenopausal and Postmenopausal Breast Cancer among Multiple Sclerosis Patients
Source: PLoS One. 2016 Oct 24;11(10):e0165027. doi: 10.1371/journal.pone.0165027 (PMC5077134; doi:10.1371/journal.pone.0165027)
Supplement: S7 Table — (DOCX) [file pone.0165027.s007.docx]

S7: Incidence rate, Hazard ratios (HR) and 95% confidence intervals (CI) for association between MS, diagnosed between 1987 and 2012, and breast cancer, stratified by menopausal status

^a^ Adjusted for age at MS diagnosis, residential location and educational level.

|  | **MS** | | | | | **Non-MS** | | | | **Unadjusted** | **Adjusted ^a^** |
| --- | --- | --- | --- | --- | --- | --- | --- | --- | --- | --- | --- |
|  | **Number** | **Person years (PY)** | **Event (%)** | **Incidence rate per 100,000 PY**  **(95% CI)** | **Number** | | **Person**  **Years (PY)** | **Event (%)** | **Incidence rate per 100,000 PY**  **(95% CI)** | **HR (95% CI)** | **HR (95% CI)** |
| **Total** | 14231 | 134378 | 257 (1.8) | 191 (169-216) | 142485 | | 1429965 | 2734 (1.9) | 191 (184-198) | 1.01 (0.89-1.15) | 1.06 (0.93-1.21) |
| **Premenopausal women** | |  |  |  |  | |  |  |  |  |  |
| **Total** | 9514 | 69681 | 58 (0.6) | 83 (64-107) | 95071 | | 698576 | 608 (0.6) | 87 (80-94) | 0.96 (0.73-1.26) | 0.96 (0.74-1.26) |
| **Age at MS diagnosis/entry** | |  |  |  |  | |  |  |  |  |  |
| <18 | 146 | 1505 | 0 (0.0) | --- | 1460 | | 15456 | 0 (0.0) | --- | --- | --- |
| 18-40 | 5597 | 51622 | 37 (0.7) | 72 (51-98) | 55914 | | 516504 | 372 (0.7) | 72 (65-80) | 1.00 (0.71-1.40) | 1.00 (0.71-1.40) |
| 41-50 | 3771 | 16555 | 21 (0.6) | 127 (81-190) | 37697 | | 166616 | 236 (0.6) | 142 (124-161) | 0.90 (0.57-1.40) | 0.90 (0.57-1.40) |
| **Postmenopausal women** | |  |  |  |  | |  |  |  |  |  |
| **Total** | 14231 | 134378 | 199 (1.4) | 148 (129-170) | 142485 | | 1429965 | 2126 (1.5) | 149 (142-155) | 1.02 (0.88-1.73) | 1.12 (0.97-1.29) |
| **Age at MS diagnosis/entry** | |  |  |  |  | |  |  |  |  |  |
| <18 | 146 | 1505 | 0 (0.0) | --- | 1460 | | 15456 | 0 (0.0) | --- | --- | --- |
| 18-40 | 5597 | 55628 | 12 (0.2) | 22 (0.12-0.37) | 55914 | | 560678 | 101 | 18 (15-22) | 1.26 (0.70-2.30) | 1.26 (0.69-2.29) |
| 41-54 | 5118 | 51702 | 99 (1.9) | 191 (1.56-2.32) | 51153 | | 543668 | 1072 | 197 (186-209) | 1.00 (0.82-1.23) | 1.00 (0.81-1.23) |
| 55-64 | 1954 | 16736 | 53 (2.7) | 317 (2.40-4.11) | 19439 | | 186726 | 583 | 312 (288-338) | 1.02 (0.77-1.35) | 1.01 (0.76-1.34) |
| ≥65 | 1416 | 8807 | 35 (2.5) | 397 (2.82-5.46) | 14519 | | 123438 | 370 | 300 (270-331) | 1.33 (0.94-1.88) | 1.31 (0.93-1.86) |
