# Supplementary material for: Risk of Premenopausal and Postmenopausal Breast Cancer among Multiple Sclerosis Patients
Source: PLoS One. 2016 Oct 24;11(10):e0165027. doi: 10.1371/journal.pone.0165027 (PMC5077134; doi:10.1371/journal.pone.0165027)
Supplement: S8 Table — (DOCX) [file pone.0165027.s008.docx]

S8: Incidence Rate, Hazard ratios (HR) and 95% confidence intervals (CI) for association between MS, diagnosed between 1987 and 2012, and breast cancer, stratified by stage of cancer and menopausal status.

^a^ Adjusted for age at MS diagnosis, residential location, duration of the MS and educational level.

|  | **MS** | | | | | **Non-MS** | | | | **Unadjusted** | **Adjusted ^a^** |
| --- | --- | --- | --- | --- | --- | --- | --- | --- | --- | --- | --- |
|  | **Number** | **Person years (PY)** | **Event (%)** | | **Incidence Rate per 100,000 PY**  **(95% CI)** | **Number** | **Person years (PY)** | **Event (%)** | **Incidence Rate per 100,000 PY**  **(95% CI)** | **HR (95% CI)** | **HR (95% CI)** |
| **Premenopausal women** | |  | | |  |  |  |  |  |  |  |
| **Total** | 9514 | 69784 | 33 (0.4) | | 47 (33-66) | 95071 | 699727 | 348 (0.4) | 50 (45-55) | 0.96 (0.67-1.36) | 0.96 (0.67-1.38) |
| Stage |  |  |  | |  |  |  |  |  |  |  |
| 0-1 | 9514 | 69849 | 14 (0.2) | | 20 (11-33) | 95071 | 700324 | 156 (0.2) | 22 (19-26) | 0.90 (0.52-1.56) | 0.92 (0.53-1.58) |
| 2 | 9514 | 69837 | 16 (0.2) | | 23 (14-36) | 95071 | 700335 | 168 (0.2) | 24 (21-28) | 0.96 (0.57-1.60) | 0.97 (0.58-1.61) |
| 3-4 | 9514 | 69876 | 3 (0.0) | | 4 (1-11) | 95071 | 700763 | 24 (0.0) | 3 (2-5) | 1.26 (0.38-4.18) | 1.26 (0.38-4.18) |
| P for Interaction |  |  |  | |  |  |  |  |  |  | 0.53 |
| **Postmenopausal women** | |  | | |  |  |  |  |  |  |  |
| **Total** | 14231 | 135341 | 108 (0.8) | 80 (66-96) | | 142485 | 1441616 | 1180 (0.8) | 82 (77-87) | 1.00 (0.82-1.22) | 1.09 (0.89-1.33) |
| Stage |  |  |  |  | |  |  |  |  |  |  |
| 0-1 | 14231 | 135483 | 65 (0.5) | 48 (37-61) | | 142485 | 1443758 | 590 (0.4) | 41 (38-44) | 1.21 (0.94-1.57) | 1.30 (1.00-1.68) |
| 2 | 14231 | 135605 | 39 (0.3) | 29 (21-39) | | 142485 | 1444180 | 503 (0.4) | 35 (32-38) | 0.85 (0.61-1.17) | 0.93 (0.67-1.29) |
| 3-4 | 14231 | 135741 | 4 (0.0) | 3 (1-7) | | 142485 | 1446129 | 87 (0.1) | 6 (5-7) | 0.52 (0.19-1.42) | 0.60 (0.22-1.63) |
| P for Interaction |  |  |  |  | |  |  |  |  |  | 0.46 |
