# Supplementary material for: Risk of Premenopausal and Postmenopausal Breast Cancer among Multiple Sclerosis Patients
Source: PLoS One. 2016 Oct 24;11(10):e0165027. doi: 10.1371/journal.pone.0165027 (PMC5077134; doi:10.1371/journal.pone.0165027)
Supplement: S9 Table — (DOCX) [file pone.0165027.s009.docx]

S9: Incidence rate, Hazard ratios (HR) and 95% confidence intervals (CI) for association between MS and breast cancer, stratified by menopausal status (follow up time since five years before the date of first registration)

^a^ Adjusted for age at MS diagnosis, residential location and educational

|  | **MS** | | | | | **Non-MS** | | | | | **Unadjusted** | **Adjusted ^a^** |  |
| --- | --- | --- | --- | --- | --- | --- | --- | --- | --- | --- | --- | --- | --- |
|  | **Number** |  | **Event (%)** |  | **Number** | |  | **Event (%)** |  | | **HR (95% CI)** | **HR (95% CI)** |  |
| **Total** | 19450 |  | 591 (3.0) |  | 194442 | |  | 6737 (3.5) |  | 0.99 (0.82-1.19) | | 1.00 (0.83-1.21) | |
| **Premenopausal women** | |  |  |  |  | |  |  |  |  | |  | |
| **Total** | 14564 |  | 123 (0.8) |  | 145420 | |  | 1270 (0.9) |  | 0.99 (0.82-1.19) | | 1.00 (0.83-1.21) | |
| **Age at MS diagnosis/entry** | |  |  |  |  | |  |  |  |  | |  | |
| <18 | 949 |  | 4 (0.4) |  | 9480 | |  | 51 (0.5) |  | 1.01 (0.13-7.81) | | 0.90 (0.11-7.00) | |
| 18-40 | 9052 |  | 82 (0.9) |  | 90451 | |  | 897 (1.0) |  | 0.89 (0.68-1.17) | | 0.89 (0.68-1.17) | |
| 41-50 | 4563 |  | 37 (0.8) |  | 45489 | |  | 322 (0.7) |  | 1.10 (0.85-1.42) | | 0.11 (0.86-1.43) | |
|  |  |  |  |  |  | |  |  |  |  | |  | |
| **Year of MS diagnosis/entry** | |  |  |  |  | |  |  |  |  | |  | |
| 1968-1980 | 2222 |  | 31 (1.4) |  | 22098 | |  | 243 (1.1) |  | 1.11 (0.93-1.97) | | 1.40 (0.96-2.03) | |
| 1981-2000 | 5325 |  | 56 (1.1) |  | 53212 | |  | 609 (1.1) |  | 0.94 (0.71-1.23) | | 0.95 (0.72-1.25) | |
| 2001-2012 | 7017 |  | 36 (0.5) |  | 70110 | |  | 418 (0.6) |  | 0.86 (0.61-1.21) | | 0.86 (0.61-1.21) | |
| **Postmenopausal women** | |  |  |  |  | |  |  |  |  | |  | |
| **Total** | 19450 |  | 468 (2.4) |  | 194442 | |  | 5467 (2.8) |  | 1.05 (0.95-1.15) | | 1.17 (1.07-1.29) | |
| **Age at MS diagnosis/entry** | |  |  |  |  | |  |  |  |  | |  | |
| <18 | 949 |  | 1 (0.1) |  | 9480 | |  | 19 (0.2) |  | ---- | | ---- | |
| 18-40 | 9052 |  | 123 (1.4) |  | 90451 | |  | 1343 (1.5) |  | 1.03 (0.80-1.33) | | 1.03 (0.80-1.32) | |
| 41-54 | 6060 |  | 199 (3.3) |  | 60447 | |  | 2555 (4.2) |  | 1.06 (0.91-1.23) | | 1.05 (0.91-1.23) | |
| 55-64 | 2024 |  | 90 (4.5) |  | 20243 | |  | 1031 (5.1) |  | 1.06 (0.88-1.28) | | 1.06 (0.88-1.27)) | |
| ≥65 | 1365 |  | 55 (4.0) |  | 13821 | |  | 519 (3.8) |  | 1.38 (1.12-1.70) | | 1.37 (1.11-1.69) | |
| **Year of MS diagnosis/entry** | |  |  |  |  | |  |  |  |  | |  | |
| 1968-1980 | 3139 |  | 133 (4.2) |  | 31297 | |  | 1854 (5.9) |  | 1.08 (0.91-1.29) | | 1.23 (1.03-1.47) | |
| 1981-2000 | 7171 |  | 214 (3.0) |  | 71768 | |  | 2541 (3.5) |  | 0.98 (0.85-1.13) | | 1.11 (0.96-1.27) | |
| 2001-2012 | 9140 |  | 121 (1.3) |  | 91377 | |  | 1072 (1.2) |  | 1.14 (0.95-1.38) | | 1.18 (0.98-1.43) | |
