# Supplementary material for: Risk of Premenopausal and Postmenopausal Breast Cancer among Multiple Sclerosis Patients
Source: PLoS One. 2016 Oct 24;11(10):e0165027. doi: 10.1371/journal.pone.0165027 (PMC5077134; doi:10.1371/journal.pone.0165027)
Supplement: S10 Table — (DOCX) [file pone.0165027.s010.docx]

S10: Incidence Rate, Hazard ratios (HR) and 95% confidence intervals (CI) for association between MS and breast cancer, stratified by stage of cancer and menopausal status, (follow up time since five years before the date of first registration)

|  | **MS** | | | | | **Non-MS** | | | | | | | **Unadjusted** | | **Adjusted ^a^** | |
| --- | --- | --- | --- | --- | --- | --- | --- | --- | --- | --- | --- | --- | --- | --- | --- | --- |
|  | **Number** |  | **Event (%)** |  | | **Number** | |  | **Event (%)** |  | | | **HR (95% CI)** | | **HR (95% CI)** | |
| **Premenopausal women** | |  | |  | |  | |  |  |  | | |  | |  | |
| **Total** | 14564 |  | 43 (0.3) |  | | 145420 | |  | 476 (0.3) |  | | | 0.93 (0.68-1.27) | | 0.95 (0.70-1.30) | |
| **Stage** |  |  |  |  | |  | |  |  |  | | |  | |  | |
| 0-1 | 14564 |  | 19 (0.1) |  | | 145420 | |  | 218 (0.2) |  | | | 0.89 (0.56-1.43) | | 0.92 (0.58-1.47) | |
| 2 | 14564 |  | 21 (0.1) |  | | 145420 | |  | 225 (0.2) |  | | | 0.96 (0.61-1.50) | | 0.98 (0.63-1.53) | |
| 3-4 | 14564 |  | 3 (0.0) |  | | 145420 | |  | 33 (0.0) |  | | | 0.94 (0.29-3.06) | | 0.94 (0.29-3.08)) | |
| P for Interaction |  |  |  |  | |  | |  |  |  | | |  | | 0.42 | |
| **Postmenopausal women** | |  | |  | |  | |  |  |  | | |  | |  | |
| **Total** | 19450 |  | 160 (0.8) | |  | | 194442 |  | 2025 (1.0) | |  | | | 1.02 (0.87-1.20) | | 1.13 (0.96-1.33) |
| **Stage** |  |  |  | |  | |  |  |  | | |  | |  | |  |
| 0-1 | 19450 |  | 93 (0.5) | |  | | 194442 |  | 994 (0.5) |  | | | | 1.20 (0.97-1.48) | | 1.30 (1.05-1.62) |
| 2 | 19450 |  | 59 (0.3) | |  | | 194442 |  | 867 (0.6) |  | | | | 0.88 (0.67-1.14) | | 0.99 (0.76-1.29) |
| 3-4 | 19450 |  | 8 (0.0) | |  | | 194442 |  | 164 (0.1) |  | | | | 0.65 (0.32-1.32) | | 0.77 (0.38-1.56) |
| P for Interaction |  |  |  | |  | |  |  |  |  | | | |  | | 0.05 |

^a^ Adjusted for age at MS diagnosis, residential location and educational level
