# Supplementary material for: Risk of Premenopausal and Postmenopausal Breast Cancer among Multiple Sclerosis Patients
Source: PLoS One. 2016 Oct 24;11(10):e0165027. doi: 10.1371/journal.pone.0165027 (PMC5077134; doi:10.1371/journal.pone.0165027)
Supplement: S11 Table — (DOCX) [file pone.0165027.s011.docx]

S11: Incidence rate, Hazard ratios (HR) and 95% confidence intervals (CI) for association between MS (Those patients who had two codes of MS) and breast cancer, stratified by menopausal status

|  | **MS** | | | | | **Non-MS** | | | | | **Unadjusted** | **Adjusted ^a^** |  |
| --- | --- | --- | --- | --- | --- | --- | --- | --- | --- | --- | --- | --- | --- |
|  | **Number** | **Person year** | **Event (%)** | **Incidence rate in 100.000 PY** | **Number** | | **Person**  **year** | **Event (%)** | **Incidence rate in 100.000 PY** | | **HR (95% CI)** | **HR (95% CI)** |  |
| **Total** | 16581 | 207291 | 404 (2.4) | 195 (177-215) | 165849 | | 2396574 | 4900 (3.0) | 204 (199-210) | 1.01 (0.91-1.11) | | 1.05 (0.95-1.17) | |
| **Premenopausal women** | |  |  |  |  | |  |  |  |  | |  | |
| **Total** | 11249 | 96860 | 77 (0.7) | 0.79 (0.63-0.99) | 112378 | | 985400 | 843 (0.8) | 0.86 (0.80-0.91) | 0.94 (0.74-1.18) | | 0.94 (0.75-1.19) | |
| **Age at MS diagnosis/entry** | |  |  |  |  | |  |  |  |  | |  | |
| <18 | 185 | 2845 | 1 (0.5) | 0.35 (0.03-1.64) | 1847 | | 29928 | 8 (0.4) | 0.27 (0.13-0.50) | 1.53 (0.19-12.25) | | 1.42 (0.18-11.46) | |
| 18-40 | 6617 | 73573 | 50 (0.8) | 0.68 (0.51-0.89) | 66068 | | 748850 | 558 (0.8) | 0.75 (0.69-0.81) | 0.93 (0.69-1.24) | | 0.93 (0.70-1.24) | |
| 41-50 | 4447 | 20442 | 26 (0.6) | 1.27 (0.85-1.83) | 44463 | | 206622 | 277 (0.6) | 1.34 (1.19-1.51) | 0.95 (0.64-1.42) | | 0.95 (0.64-1.43) | |
|  |  |  |  |  |  | |  |  |  |  | |  | |
| **Year of MS diagnosis/entry** | |  |  |  |  | |  |  |  |  | |  | |
| 1968-1980 | 1582 | 20334 | 14 (0.9) | 0.69 (0.39-1.12) | 15796 | | 215617 | 164 (1.0) | 0.76 (0.65-0.88) | 0.93 (0.54-1.60) | | 0.95 (0.55-1.63) | |
| 1981-2000 | 4147 | 47462 | 41 (1.0) | 0.86 (0.63-1.16) | 41461 | | 481042 | 464 (1.1) | 0.96 (0.88-1.06) | 0.90 (0.65-1.24) | | 0.91 (0.66-1.25) | |
| 2001-2012 | 5520 | 29064 | 22 (0.4) | 0.76 (0.49-1.13) | 55121 | | 288741 | 215 (0.4) | 0.74 (0.65-0.85) | 1.02 (0.66-1.58) | | 1.02 (0.66-1.58) | |
| **Postmenopausal women** | |  |  |  |  | |  |  |  |  | |  | |
| **Total** | 16581 | 207291 | 327 (2.0) | 1.58 (1.41-1.76) | 165849 | | 2396574 | 4057 (2.5) | 1.69 (1.64-1.75) | 1.01 (0.90-1.13) | | 1.11 (0.99-1.24) | |
| **Age at MS diagnosis/entry** | |  |  |  |  | |  |  |  |  | |  | |
| <18 | 185 | 2884 | 0 (0.0) | 0 (0.0) | 1847 | | 30656 | 0 (0.0) | 0 (0.0) | ---- | | ---- | |
| 18-40 | 6617 | 91447 | 53 (0.8) | 0.58 (0.44-0.75) | 66068 | | 983697 | 699 (1.1) | 0.71 (0.66-0.76) | 0.98 (0.74-1.30) | | 0.98 (0.74-1.30) | |
| 41-54 | 6055 | 76364 | 156 (2.6) | 2.04 (1.74-2.38) | 60482 | | 907768 | 1945 (3.2) | 2.14 (2.05-2.24) | 1.05 (0.89-1.23) | | 1.04 (0.89-1.23) | |
| 55-64 | 2371 | 25785 | 74 (3.1) | 2.87 (2.27-3.58) | 23625 | | 326826 | 973 (4.1) | 2.98 (2.79-3.17) | 0.98 (0.78-1.25) | | 0.98 (0.78-1.25) | |
| ≥65 | 1353 | 10811 | 44 (3.3) | 4.07 (3.00-5.41) | 13827 | | 147626 | 440 (3.2) | 2.98 (2.71-3.27) | 1.40 (1.02-1.91) | | 1.39 (1.02-1.90) | |
| **Year of MS diagnosis/entry** | |  |  |  |  | |  |  |  |  | |  | |
| 1968-1980 | 2506 | 53882 | 102 (4.1) | 1.89 (1.55-2.29) | 24999 | | 738612 | 1479 (5.9) | 2.00 (1.90-2.11) | 1.07 (0.87-1.30) | | 1.19 (0.97-1.46) | |
| 1981-2000 | 6133 | 103526 | 161 (2.6) | 1.56 (1.33-1.81) | 61384 | | 1155636 | 1959 (3.2) | 1.70 (1.62-1.77) | 0.95 (0.81-1.12) | | 1.05 (0.89-1.23) | |
| 2001-2012 | 7942 | 49882 | 64 (0.8) | 1.28 (1.00-1.63) | 79466 | | 502325 | 619 (0.8) | 1.23 (1.14-1.33) | 1.04 (0.81-1.35) | | 1.07 (0.83-1.39) | |

^a^ Adjusted for age at MS diagnosis, residential location and educational level
